# Enhanced Expression of WD Repeat-Containing Protein 35 via CaMKK/AMPK Activation in Bupivacaine-Treated Neuro2a Cells

**DOI:** 10.1371/journal.pone.0098185

**Published:** 2014-05-23

**Authors:** Lei Huang, Fumio Kondo, Masahiko Gosho, Guo-Gang Feng, Misako Harato, Zhong-yuan Xia, Naohisa Ishikawa, Yoshihiro Fujiwara, Shoshiro Okada

**Affiliations:** 1 Department of Pharmacology, Aichi Medical University School of Medicine, Nagakute, Aichi, Japan; 2 Advanced Medical Research Center, Aichi Medical University, Nagakute, Aichi, Japan; 3 Department of Anesthesiology, Aichi Medical University School of Medicine, Nagakute, Aichi, Japan; 4 Department of Anesthesiology, Renmin Hospital of Wuhan University, Wuhan, Hubei, China; University of Missouri-Kansas City, United States of America

## Abstract

We previously reported that bupivacaine induces reactive oxygen species (ROS) generation, p38 mitogen-activated protein kinase (MAPK) activation and nuclear factor-kappa B activation, resulting in an increase in expression of WD repeat-containing protein 35 (WDR35) in mouse neuroblastoma Neuro2a cells. However, the identity of signaling upstream of p38 MAPK pathways to WDR35 expression remains unclear. It has been shown that AMP-activated protein kinase (AMPK) can activate p38 MAPK through diverse mechanisms. In addition, several kinases acting upstream of AMPK have been identified including Ca^2+^/calmodulin-dependent protein kinase kinase (CaMKK). Recent studies reported that AMPK may be involved in bupivacaine-induced cytotoxicity in Schwann cells and in human neuroblastoma SH-SY5Y cells. The present study was undertaken to test whether CaMKK and AMPK are involved in bupivacaine-induced WDR35 expression in Neuro2a cells. Our results showed that bupivacaine induced activation of AMPK and p38 MAPK in Neuro2a cells. The AMPK inhibitors, compound C and iodotubercidin, attenuated the bupivacaine-induced activation of AMPK and p38 MAPK, resulting in an inhibition of the bupivacaine-induced increase in WDR35 expression. Treatment with the CaMKK inhibitor STO-609 also attenuated the bupivacaine-induced activation of AMPK and p38 MAPK, resulting in an inhibition of the bupivacaine-induced increase in WDR35 expression. These results suggest that bupivacaine activates AMPK and p38 MAPK via CaMKK in Neuro2a cells, and that the CaMKK/AMPK/p38 MAPK pathway is involved in regulating WDR35 expression.

## Introduction

The family of WD repeat (WDR) proteins comprises a large number of proteins and is involved in a wide variety of cellular processes such as signal transduction, cell growth, proliferation, and apoptosis [1], [2]. WD repeat-containing protein 35 (WDR35) is a novel member of the WDR protein family [3]. Previously, we reported that enhanced WDR35 expression may mediate apoptosis in several animal models [4]–[6].

Bupivacaine-induced neurotoxicity has been associated with the generation of reactive oxygen species (ROS) [7] and activation of p38 mitogen-activated protein kinase (MAPK) [8], [9]. Recently, we demonstrated that bupivacaine induces ROS generation and p38 MAPK activation, resulting in an increase in WDR35 expression in mouse neuroblastoma Neuro2a cells [10]. More recently, we reported that bupivacaine induces the activation of nuclear factor-kappa B (NF-κB) in Neuro2a cells, and activation of NF-κB is involved in the bupivacaine-induced increase in WDR35 expression [11]. However, the identity of signaling upstream of p38 MAPK pathways to WDR35 expression remains unclear.

Many studies have demonstrated that AMP-activated protein kinase (AMPK) can activate p38 MAPK through diverse mechanisms [12]–[14]. AMPK is a heterotrimeric enzyme consisting of catalytic α- and regulatory β- and γ- subunits. Activation of AMPK requires phosphorylation of threonine (Thr172) in the activation loop of the α-subunit by upstream kinases [15]–[17]. AMPK is considered to be a regulator of cellular energy homeostasis, whereby it senses the metabolic status within a cell, especially under ATP deprivation, and is associated with the regulation of cellular stress in various cell types [17]–[19]. Recent studies demonstrated that AMPK may be involved in bupivacaine-induced cytotoxicity in Schwann cells [20] and in human neuroblastoma SH-SY5Y cells [21]. Several kinases acting upstream of AMPK have been identified including Ca^2+^/calmodulin-dependent protein kinase kinase (CaMKK), which can activate AMPK by phosphorylating the α-subunit at Thr172 [22], [23]. Recently, Pfisterer et al. [24] reported that CaMKK signaling via AMPK contributes to the regulation of WD-repeat protein interacting with phosphoinositides (WIPI)-1, another WDR protein family member, in starvation-induced autophagy. However, the involvement of CaMKK and AMPK to WDR35 expression has not been investigated. The present study was undertaken to test whether CaMKK and AMPK are involved in bupivacaine-induced WDR35 expression in Neuro2a cells. Our results suggest that AMPK is activated by bupivacaine in Neuro2a cells, and that the CaMKK/AMPK/p38 MAPK pathway is involved in regulating WDR35 expression.

## Materials and Methods

### Cell culture

Mouse neuroblastoma Neuro2a cells were purchased from the Health Science Research Resources Bank (Tokyo, Japan). The cells were maintained in RPMI-1640 medium (Sigma-Aldrich, St. Louis, MO, USA) containing 10% fetal bovine serum with 100 units/ml penicillin and 100 g/ml streptomycin (Gibco BRL, Grand Island, NY, USA). The cells were maintained at 37°C in a humidified atmosphere with 5% CO_2_. The culture medium was replaced every 2–3 days. To prepare cell suspensions, the cells were treated with trypsin (0.25%)-EDTA (1 mM) (Gibco BRL, Grand Island, NY, USA), transferred to a 6-cm culture plate at a density of 1.5×10^6^ cells per dish, and cultured overnight.

In our previous study, we reported that bupivacaine dose-dependently increased WDR35 expression and that maximal WDR35 expression was observed with a concentration of 2 mM bupivacaine at 9 h [10]. As the maximal effect was reached at 9 h, the same 9 h time point of 2 mM bupivacaine treatment was used in the present study.

### Measurement of ATP levels

Intracellular ATP levels were measured with a CellTiter-Glo Luminescent Cell Viability Assay kit (Promega, Madison, WI) according to the manufacturer's instructions. Briefly, after exposure of the cells to 2 mM bupivacaine for a period of 1 to 9 h, 100 µl of CellTiter-Glo reagent was added to 100 µl of the cell-containing medium. The contents were then mixed for 2 min to induce cell lysis, and the cells were incubated at room temperature for an additional 10 min to stabilize the luminescent signal. The luminance (RLU) was measured with a microplate reader (VersaMax, Molecular Devices, Sunnyvale, CA, USA).

### Western blot analysis

Protein samples from cultured Neuro2a cells were homogenized in sample buffer [50 mM Tris-HCl (pH 6.8), 0.2 M DTT, 2% sodium dodecyl sulfate (SDS), 10% glycerol, 0.1% bromophenol blue (BPB)] containing a mixture of protease inhibitors (Complete Protease Inhibitor Cocktail, Roche Applied Sciences, Mannheim, Germany) and heated in boiling water for 5 min. Proteins were separated by SDS-PAGE and transferred to PVDF membranes (Immobilon-P, Millipore, Bedford, MA, USA). The membranes were blocked in 5% w/v bovine serum albumin in Tris-buffered saline (TBST, 0.05 M Tris, 0.15 M NaCl, 0.1% Tween-20, pH 7.4) overnight at 4°C. These membranes were probed with an anti-WDR35 peptide antibody (amino acids 459-473, 1∶500), which was designed, produced, and purified by Medical & Biological Laboratories (Nagoya, Japan), or with antibodies against AMPKα, phospho-AMPKα (Thr172) (P-AMPKα), p38 MAPK, phospho-p38 (P-p38) MAPK, or β-actin (Cell Signaling Technology, Danvers, MA, USA; 1∶1000). Detection was performed with the Western blotting reagent ECL Prime (GE Healthcare, Buckinghamshire, UK). Protein levels were quantified by densitometric scanning with the Gel-Pro Analyzer (Media Cybernetics, Inc., USA) and expressed as the ratio to β-actin levels as described previously [10], [11].

### Quantitative real-time polymerase chain reaction (qPCR) analysis

Total RNA (1 µg) was extracted from cultured Neuro2a cells with TRIzol reagent (Invitrogen, Carlsbad, CA, USA) and reverse transcribed with the ReverTra Ace qPCR RT kit (Toyobo, Osaka, Japan). qPCR was performed with the ABI StepOne Plus real-time PCR system and a TaqMan Gene Expression Assay (Applied Biosystems, Tokyo, Japan) according to the manufacturer's instructions. The primers and TaqMan MGB probe for mouse WDR35 (Mm00552650_m1) were purchased from Applied Biosystems. The amount of WDR35 PCR product was calculated relative to the internal controls β-actin (ACTB, Mm00607939_s1; Applied Biosystems), glyceraldehyde-3-phosphate dehydrogenase (GAPDH, Mm99999915_g1), or 18S ribosomal RNA (18S rRNA, Mm03928990_g1), and was compared between experimental and control groups by the ΔΔC_T_ method, as reported previously [10], [11]. The difference in internal controls did not affect the results in the qPCR experiments.

### Statistical analysis

All results are expressed as the mean ± standard error of the mean (SEM). Data were analyzed by one-way or two-way analysis of variance (ANOVA) followed by Tukey's post-hoc test. Statistical analysis for time effect was performed by repeated measures one-way ANOVA. Treatment and time effects were analyzed using repeated measures two-way ANOVA including treatment group and time period as factors and the interaction term between treatment group and time period. Differences were considered significant at *P*<0.05. All statistical analyses were done with the SAS 9.3 (SAS institute, Cary, USA).

## Results

### Bupivacaine induces AMPK activation in Neuro2a cells

**Figure 1:**
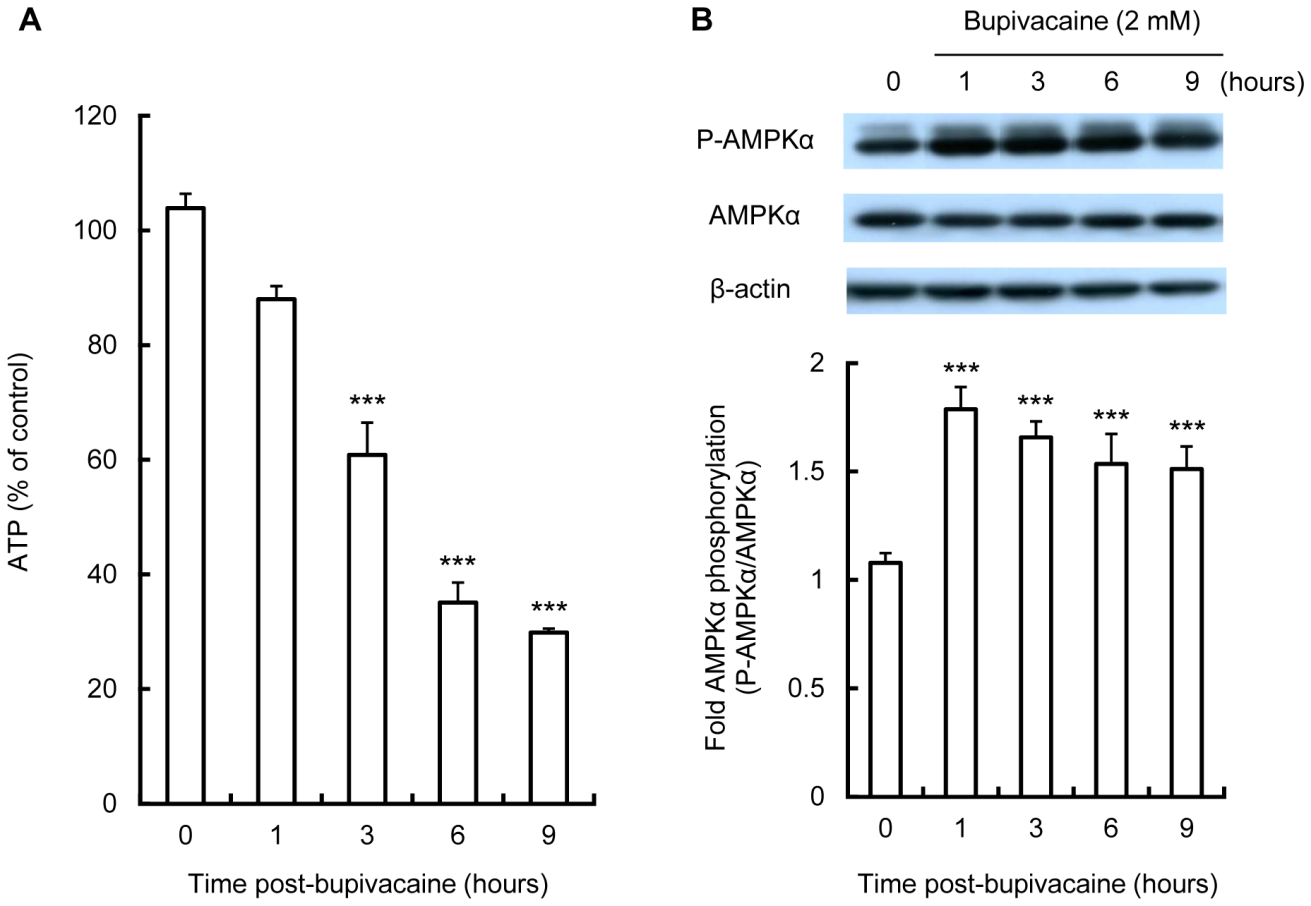
Effect of bupivacaine on intracellular ATP levels and AMPK activation. Neuro2a cells were treated with 2(A) Intracellular levels of ATP were measured with a luminescence assay (n = 4 per group). (B) Expression of phospho-AMPKα (P-AMPKα) and AMPK following cell exposure to bupivacaine was measured by Western blotting (n = 3 per group). ***P<0.001 vs. control (not treated with bupivacaine). All post hoc pairwise comparisons were performed by Tukey's test with repeated measures one-way ANOVA.

We first measured the intracellular ATP levels in bupivacaine-treated Neuro2a cells. As shown in Figure 1A, treatment with 2 mM bupivacaine significantly reduced intracellular ATP levels in Neuro2a cells in a time-dependent manner (*P*<0.001 at 3 h and later; Figure 1A).

To determine whether AMPK is activated in bupivacaine-treated Neuro2a cells, we then examined AMPKα and phospho-AMPKα (P-AMPKα) protein expression in bupivacaine-treated Neuro2a cells by Western blotting and expressed the results as the P-AMPKα/AMPKα ratio. As shown in Figure 1B, treatment of Neuro2a cells with 2 mM bupivacaine significantly increased phosphorylation levels of AMPKα at 1 to 9 h (*P*<0.001 at 1 h and later).

### AMPK inhibitors, compound C and iodotubercidin, attenuate the bupivacaine-induced increase in AMPK and p38 MAPK activity in Neuro2a cells

**Figure 2:**
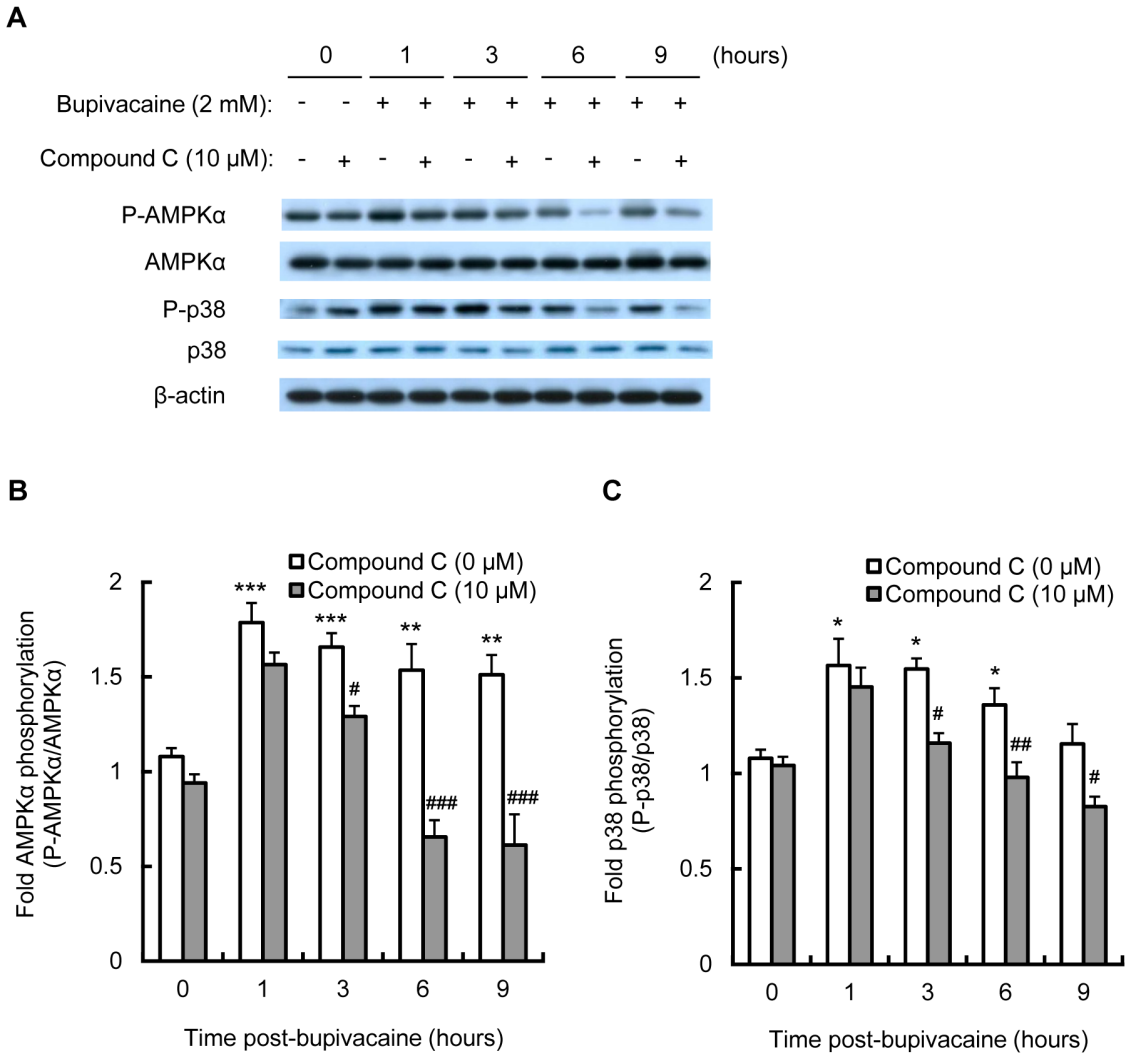
Effect of compound C on bupivacaine-induced AMPK and p38 MAPK activity. Neuro2a cells were treated with AMPK inhibitor compound C (10 µM) for 1 h, followed by bupivacaine (2 mM) for 9 h. (A) Expression of phospho-AMPKα (P-AMPKα), AMPK, phospho-p38 (P-p38) and p38 was measured by Western blotting (n = 3 per group). The bar diagram shows the ratio of phospho-AMPKα to AMPK (B) and the ratio of P-p38 to p38 (C). *P<0.05, **P<0.01 and ***P<0.001 vs. control (not treated with bupivacaine), ^#^ *P*<0.05, ^##^ *P*<0.01 and ^###^ *P*<0.001 vs. absence of compound C. All post hoc comparisons were performed by Tukey's test with repeated measures two-way ANOVA including compound C group and time period as factors and the interaction term between the two factors.

In order to explore the relationship between AMPK and p38 MAPK activation in bupivacaine-treated Neuro2a cells, cells were treated for 1 h with the AMPK inhibitor compound C (10 µM; Calbiochem, La Jolla, CA, USA) or iodotubercidin (1 µM; Abcam, Cambridge, MA, USA), followed by bupivacaine (2 mM) for 1 to 9 h. As shown in Figure 2A and 2B, compound C significantly attenuated the bupivacaine-induced increase in phosphorylation levels of AMPKα at time points from 3 to 9 h (*P*<0.05 and *P*<0.001). Compound C also significantly attenuated the bupivacaine-induced increase in phosphorylation levels of p38 MAPK at time points from 3 to 9 h (*P*<0.05 and *P*<0.01; Figure 2A and 2C).

**Figure 3:**
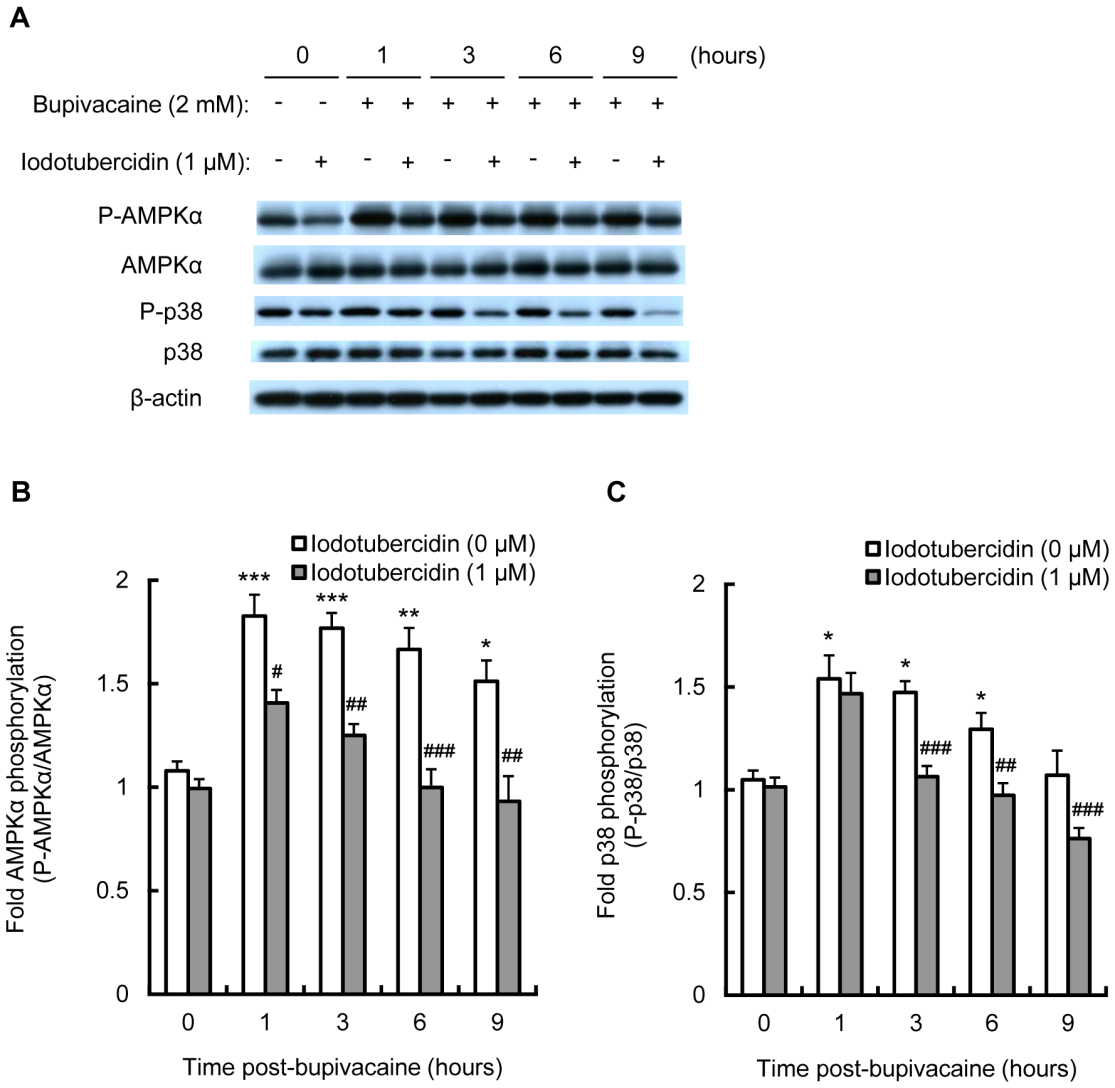
Effect of iodotubercidin on bupivacaine-induced AMPK and p38 MAPK activity. Neuro2a cells were treated with AMPK inhibitor iodotubercidin (1 µM) for 1 h, followed by bupivacaine (2 mM) for 9 h. (A) Expression of phospho-AMPKα (P-AMPKα), AMPK, phospho-p38 (P-p38) and p38 was measured by Western blotting (n = 3 per group). The bar diagram shows the ratio of phospho-AMPKα to AMPK (B) and the ratio of P-p38 to p38 (C). *P<0.05, **P<0.01 and ***P<0.001 vs. control (not treated with bupivacaine), ^#^ *P*<0.05, ^##^ *P*<0.01 and ^###^ *P*<0.001 vs. absence of iodotubercidin. All post hoc comparisons were performed by Tukey's test with repeated measures two-way ANOVA including iodotubercidin group and time period as factors and the interaction term between the two factors.

Treatment with iodotubercidin significantly attenuated the bupivacaine-induced increase in phosphorylation levels of AMPKα at time points from 1 to 9 h (*P*<0.05, *P*<0.01 and *P*<0.001; Figure 3A and 3B). Iodotubercidin also significantly attenuated the bupivacaine-induced increase in phosphorylation levels of p38 MAPK at time points from 3 to 9 h (*P*<0.01 and *P*<0.001; Figure 3A and 3C).

### AMPK inhibitors, compound C and iodotubercidin, attenuate the bupivacaine-induced increase in WDR35 expression in Neuro2a cells

**Figure 4:**
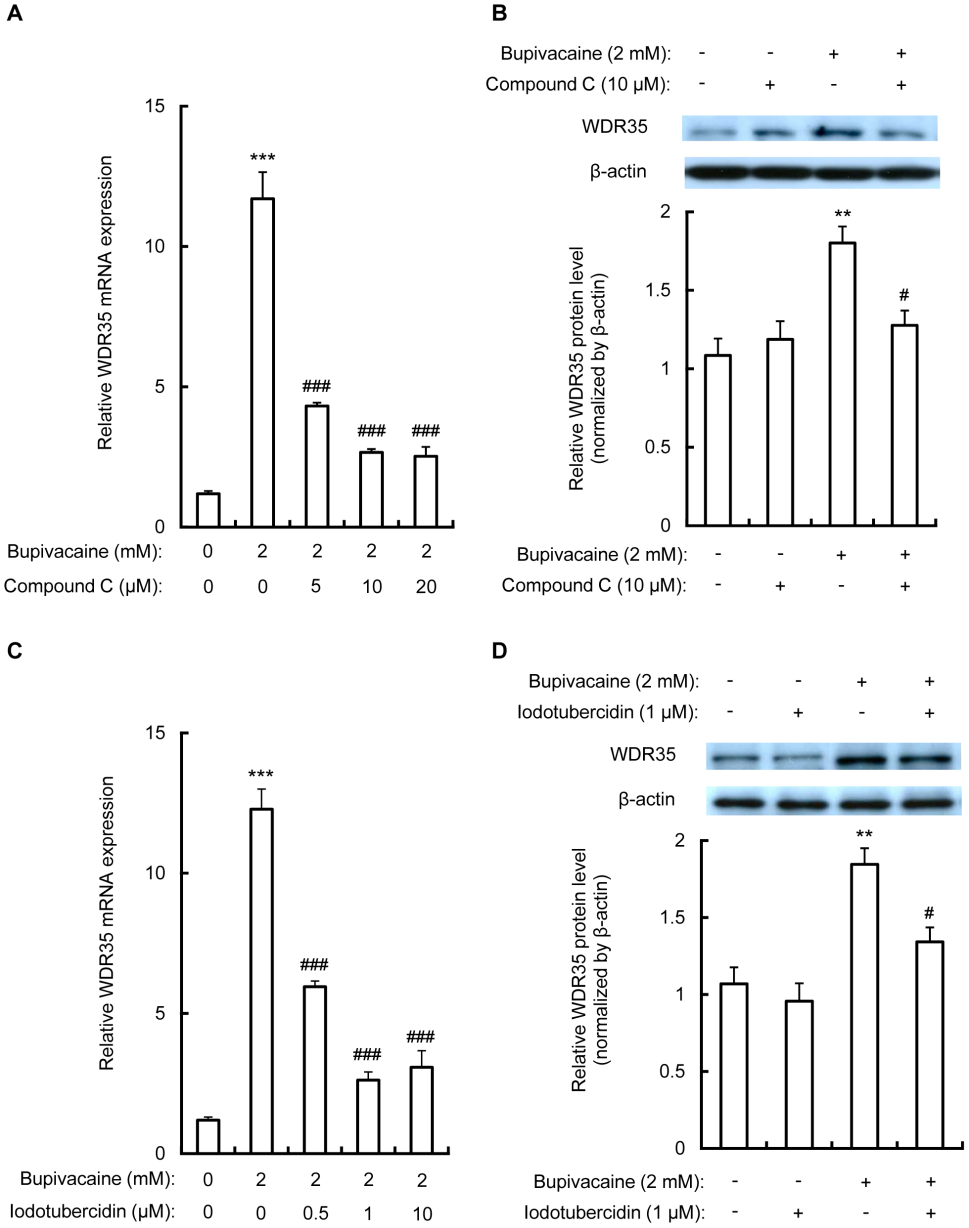
Effect of compound C and iodotubercidin on bupivacaine-induced WDR35 expression. (A) Neuro2a cells were treated with various concentrations of compound C for 1 h, followed by bupivacaine (2 mM) for 9 h. WDR35 mRNA expression was analyzed by qPCR and expressed relative to the level of β-actin mRNA (n = 4 per group). All post hoc comparisons were performed by Tukey's test with one-way ANOVA. (B) WDR35 protein expression with or without 10 µM compound C was analyzed by Western blotting (n = 4 per group). All post hoc comparisons were performed by Tukey's test with two-way ANOVA including bupivacaine and compound C groups as factors and the interaction term between the two factors. (C) Cells were treated with various concentrations of iodotubercidin for 1 h, followed by bupivacaine (2 mM) for 9 h. WDR35 mRNA expression was analyzed by qPCR (n = 4 per group). All post hoc comparisons were performed by Tukey's test with one-way ANOVA. (D) WDR35 protein expression with or without 1 µM iodotubercidin was analyzed by Western blotting (n = 3 per group). All post hoc comparisons were performed by Tukey's test with two-way ANOVA including bupivacaine and iodotubercidin groups as factors and the interaction term between the two factors. **P<0.01 and ***P<0.001 vs. control (not treated with bupivacaine), ^#^ *P*<0.05 and ^###^ *P*<0.001 vs. treatment with bupivacaine alone.

In order to explore the relationship between WDR35 expression and AMPK activation, Neuro2a cells were treated for 1 h with compound C (5, 10 and 20 µM) or iodotubercidin (0.5, 1 and 10 µM), followed by bupivacaine (2 mM) for 9 h. As shown in Figure 4A, compound C significantly attenuated the bupivacaine-induced increase in WDR35 mRNA expression in a dose-dependent manner (*P*<0.001). Treatment with 10 µM compound C significantly attenuated the bupivacaine-induced increase in WDR35 protein expression (*P*<0.05; Figure 4B).

Treatment with iodotubercidin significantly attenuated the bupivacaine-induced increase in WDR35 mRNA expression in a dose-dependent manner (*P*<0.001; Figure 4C). Iodotubercidin also significantly attenuated the bupivacaine-induced increase in WDR35 protein expression (*P*<0.05; Figure 4D).

### STO-609, a CaMKK inhibitor, attenuates the bupivacaine-induced increase in AMPK and p38 MAPK activity in Neuro2a cells

**Figure 5:**
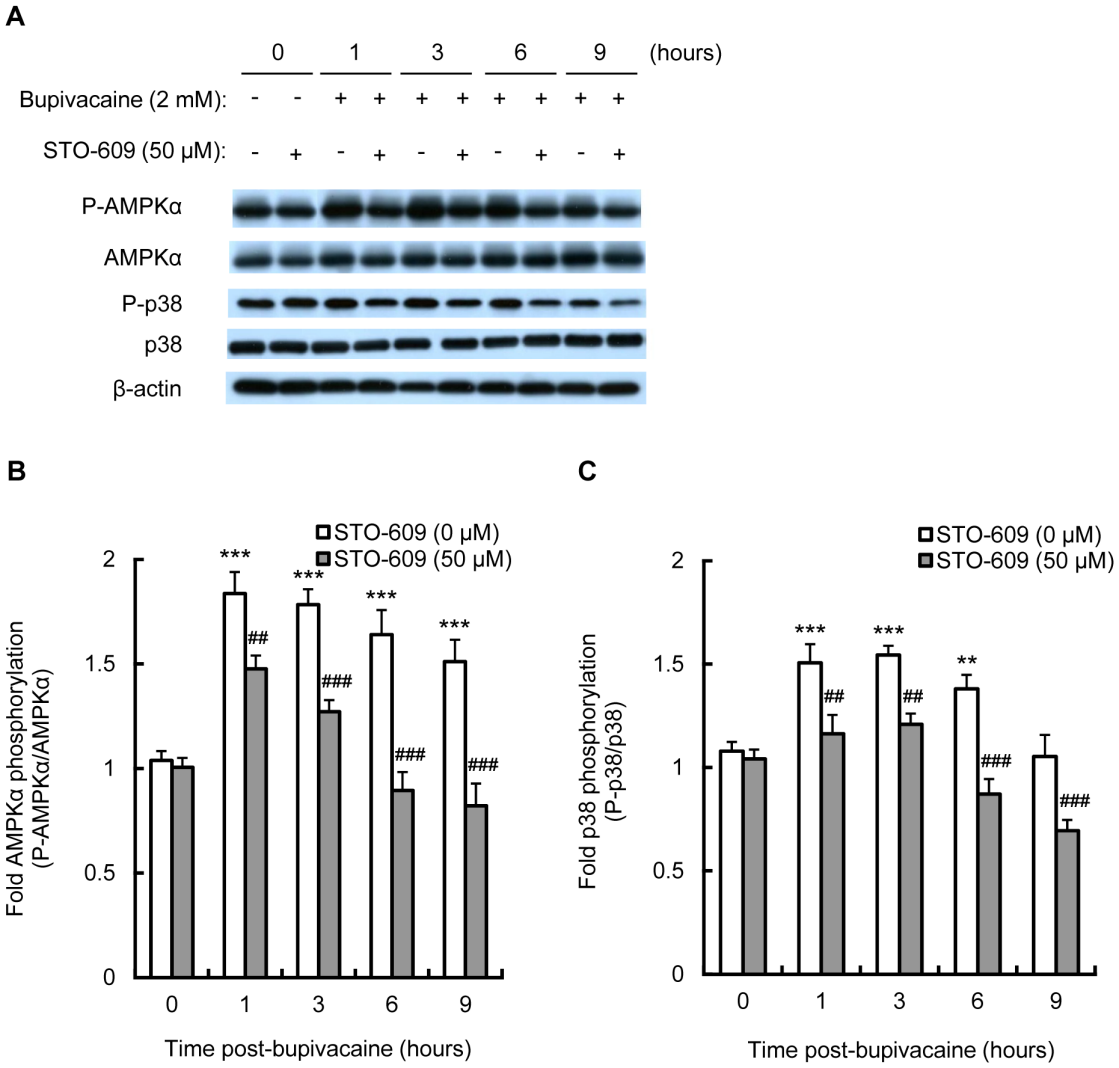
Effect of STO-609 on bupivacaine-induced AMPK and p38 MAPK activity. Neuro2a cells were treated with CaMKK inhibitor STO-609 (50 µM) for 1 h, followed by bupivacaine (2 mM) for 9 h. (A) Expression of phospho-AMPKα (P-AMPKα), AMPK, phospho-p38 (P-p38) and p38 was measured by Western blotting (n = 3 per group). The bar diagram shows the ratio of phospho-AMPKα to AMPK (B) and the ratio of P-p38 to p38 (C). **P<0.01 and ***P<0.001 vs. control (not treated with bupivacaine), ^##^ *P*<0.01 and ^###^ *P*<0.001 vs. absence of STO-609. All post hoc comparisons were performed by Tukey's test with repeated measures two-way ANOVA including STO-609 group and time period as factors and the interaction term between the two factors.

Several kinases acting upstream of AMPK have been identified including CaMKK which is highly expressed in neurons [25]–[27]. In order to examine whether CaMKK is involved in bupivacaine-induced AMPK activation, we further investigated the effects of STO-609, a CaMKK inhibitor, on AMPKα protein expression in bupivacaine-treated Neuro2a cells. Neuro2a cells were treated with STO-609 (50 µM; Calbiochem, La Jolla, CA, USA) for 1 h, followed by bupivacaine (2 mM) for 1 to 9 h. As shown in Figure 5A and 5B, treatment with STO-609 significantly attenuated the bupivacaine-induced increase in phosphorylation levels of AMPKα at time points from 1 to 9 h (*P*<0.01 and *P*<0.001). Similarly, STO-609 significantly attenuated the bupivacaine-induced increase in phosphorylation levels of p38 MAPK at time points from 1 to 9 h (*P*<0.01 and *P*<0.001; Figure 5A and 5C).

### STO-609 attenuates the bupivacaine-induced increase in WDR35 expression in Neuro2a cells

**Figure 6:**
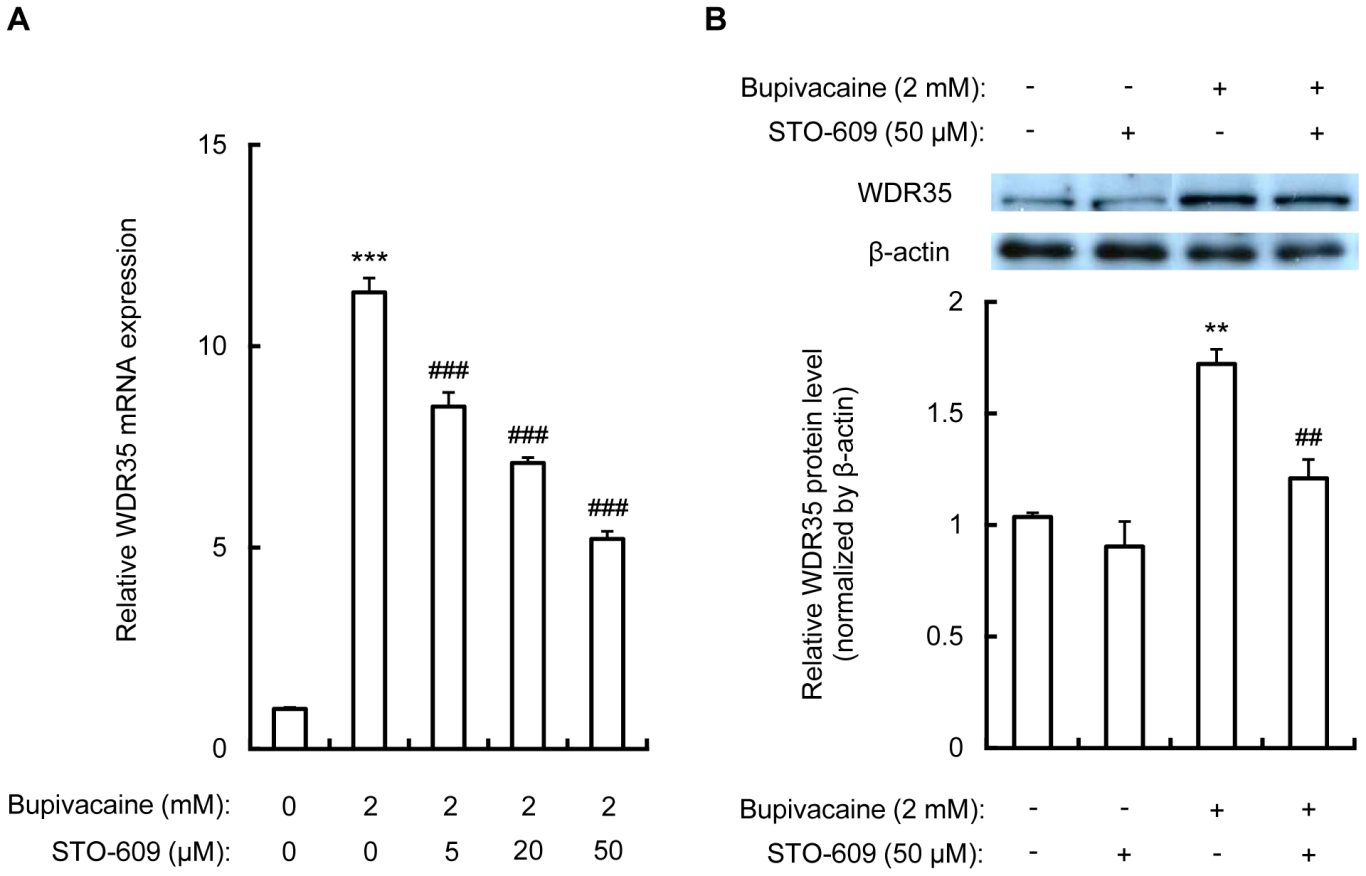
Effect of STO-609 on bupivacaine-induced WDR35 expression. (A) Neuro2a cells were treated with various concentrations of STO-609 for 1 h, followed by bupivacaine (2 mM) for 9 h. WDR35 mRNA expression was analyzed by qPCR and expressed relative to the level of β-actin mRNA (n = 4 per group). All post hoc comparisons were performed by Tukey's test with one-way ANOVA. (B) WDR35 protein expression with or without 50 µM STO-609 was analyzed by Western blotting (n = 3 per group). All post hoc comparisons were performed by Tukey's test with two-way ANOVA including bupivacaine and STO-609 groups as factors and the interaction term between the two factors. **P<0.01 and ***P<0.001 vs. control (not treated with bupivacaine), ^##^ *P*<0.01 and ^###^ *P*<0.001 vs. absence of STO-609.

In order to explore the relationship between WDR35 expression and CaMKK, Neuro2a cells were treated for 1 h with STO-609 (5, 20 and 50 µM), followed by bupivacaine (2 mM) for 9 h. As shown in Figure 6A, STO-609 significantly attenuated the bupivacaine-induced increase in WDR35 mRNA expression in a dose-dependent manner (*P*<0.001). In addition, treatment with 50 µM STO-609 significantly attenuated the bupivacaine-induced increase in WDR35 protein expression (*P*<0.01; Figure 6B).

## Discussion

We previously reported that bupivacaine induces ROS generation, p38 MAPK activation and NF-κB activation, resulting in an increase in expression of WDR35 in Neuro2a cells [10], [11]. In the present study, we investigated the relevance of kinases acting upstream of p38 MAPK to WDR35 expression. Bupivacaine induced activation of AMPK and p38 MAPK in Neuro2a cells. The AMPK inhibitors, compound C and iodotubercidin, attenuated the bupivacaine-induced activation of AMPK and p38 MAPK, resulting in an inhibition of the bupivacaine-induced increase in WDR35 expression. Treatment with the CaMKK inhibitor STO-609 also attenuated the bupivacaine-induced activation of AMPK and p38 MAPK, resulting in an inhibition of the bupivacaine-induced increase in WDR35 expression. Collectively, these results suggest that the CaMKK/AMPK/p38 MAPK pathway is involved in regulating WDR35 expression in bupivacaine-treated Neuro2a cells.

At the molecular level, AMPK is considered to regulate energy balance in response to decreases in ATP, which is important for regulating cellular homeostasis under various kinds of stress [17], [22], [23]. In the present study, we showed that 2 mM bupivacaine reduced intracellular ATP levels in a time-dependent manner in Neuro2a cells. Our results are consistent with a previous report that showed the same dose of bupivacaine decreased intracellular ATP levels in B16 melanoma cells [28]. It has been reported that bupivacaine increased AMPK activity in Schwann cells [20], and we subsequently confirmed the activation of AMPK in bupivacaine-treated Neuro2a cells. These results demonstrated that bupivacaine reduced intracellular ATP levels and increased AMPK activity in Neuro2a cells.

In the present study, we showed that the AMPK inhibitors, compound C and iodotubercidin, attenuated the bupivacaine-induced increase in AMPK and p38 MAPK activity in Neuro2a cells. These results are in agreement with previous reports that AMPK activation could lead to p38 MAPK activation in airway epithelial cells [12], osteoblasts [13] and muscle cells [14]. CaMKK, one of the upstream AMPK kinases, is abundantly expressed in neurons and is activated by an increase in intracellular Ca^2+^ concentration [25]–[27]. In neurons, Ca^2+^ balance dysfunction has been shown to trigger AMPK signaling [23]. Recently, Wen et al. [29] reported that bupivacaine treatment leads to an increase in intracellular Ca^2+^ concentration in SH-SY5Y cells. We thus examined the effects of STO-609, a CaMKK inhibitor, on AMPK and p38 MAPK activity. STO-609 treatment attenuated the bupivacaine-induced increase in AMPK and p38 MAPK activity. Collectively, these results indicate that CaMKK is involved in bupivacaine-induced AMPK and p38 MAPK activation.

Recently, we demonstrated that bupivacaine induces ROS generation and p38 MAPK activation, resulting in an increase in WDR35 expression in Neuro2a cells [10]. To determine whether AMPK activation is involved in bupivacaine-induced WDR35 expression, we examined the effects of the AMPK inhibitors, compound C and iodotubercidin, on WDR35 expression in bupivacaine-treated Neuro2a cells. These inhibitors significantly attenuated the bupivacaine-induced increase in WDR35 expression in Neuro2a cells. Moreover, a separate research group reported that CaMKK signaling via AMPK contributes to the regulation of another WDR protein, WIPI-1 [24]. Here, we showed that the CaMKK inhibitor, STO-609, attenuated the bupivacaine-induced increase in WDR35 expression in Neuro2a cells. Collectively, these results indicate that the CaMKK/AMPK/p38 MAPK pathway is involved in regulating WDR35 expression in bupivacaine-treated Neuro2a cells.

The STO-609 treatment only partially altered the transcriptional activation of WDR35 while potently inhibited AMPK and p38 MAPK, a downstream target of CaMKK. This result is surprising since acting more downstream in the cascade is supposed to elicit less dramatic effects on the activation of transcriptional programs. Although compound C and STO-609 have been shown to have some “off target” effects [30], these findings raise an interesting possibility that another signaling pathway might be involved in the induction of WDR35 in response to bupivacaine.

Recent studies have demonstrated that the *WDR35* gene is involved in several human diseases such as type 2 diabetes [31], acute lymphoblastic leukemia [32], coronary artery disease [33], and Sensenbrenner syndrome [34]. However, the regulation of WDR35 expression by intracellular signaling pathways has not been fully elucidated. In the present study, we provide the first evidence of the sequence of events in bupivacaine-induced WDR35 expression by *in vitro* biochemical and pharmacological examinations, and show that CaMKK/AMPK signaling acts upstream to regulate WDR35 expression. Since the function of WDR35 is still unknown, further studies incorporating (1) proteome analysis of proteins interacting with WDR35 and (2) promoter analysis of the *WDR35* gene will help to understand the role of WDR35.

**Figure 7:**
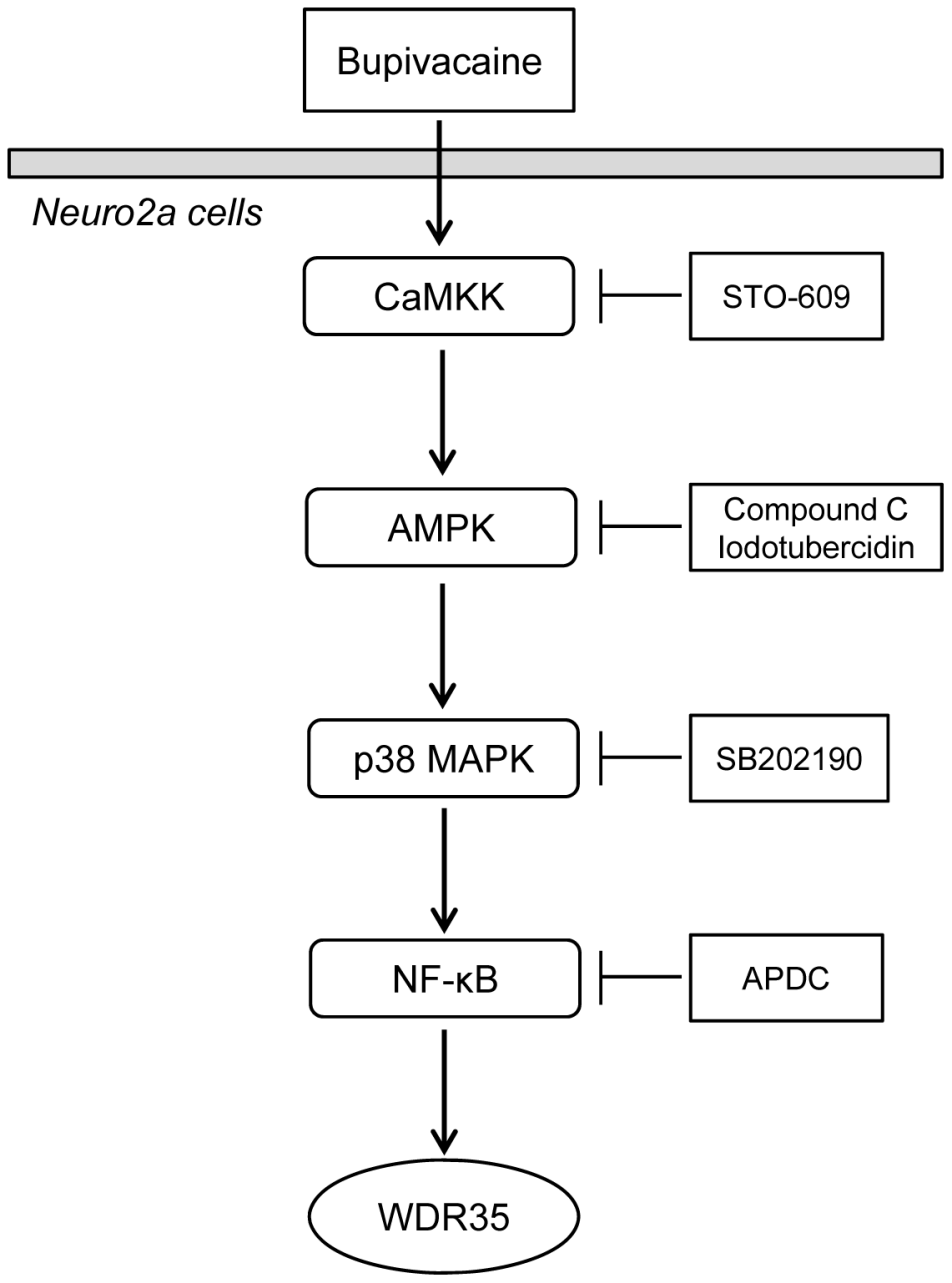
Schematic diagram illustrating the proposed signaling pathway involved in bupivacaine-induced WDR35 expression in Neuro2a cells.

In conclusion, our results indicate that bupivacaine activates AMPK and p38 MAPK via CaMKK in Neuro2a cells. Taken together with our previous study, the CaMKK/AMPK/p38 MAPK/NF-κB pathway is involved in regulating WDR35 expression in bupivacaine-treated Neuro2a cells (Figure 7).
