# Expression of Concern: Enhanced Expression of WD Repeat-Containing Protein 35 via CaMKK/AMPK Activation in Bupivacaine-Treated Neuro2a Cells

**DOI:** 10.1371/journal.pone.0213041

**Published:** 2019-02-22

**Authors:** The PLOS ONE Editors

Following the publication of [1] concerns were raised in the following figure panels:

- Fig 1B AMPKα panel appears similar to Fig 2A AMPKα panel
- Fig 1B P-AMPKα panel contains vertical discontinuities across lanes
- Fig 3A P-AMPKα panel contains vertical discontinuities across lanes
- Fig 3A P-p38 panel contains a vertical discontinuity after lane 9
- Fig 5A P-AMPKα panel contains a vertical discontinuity after lane 2
- Fig 6B WDR35 panel contains a vertical discontinuity after lane 2

The authors explained that an incorrect image was used during preparation of Fig 1 and have provided a replacement image for the Fig 1B AMPKα panel. In the case of the Fig 1B P-AMPKα panel, the authors explained that samples were run in duplicate for Bupivacaine treatment and they removed the duplicate samples’ data in preparing the figure. However, the raw data provided by the authors suggests that the results across these duplicate samples were variable and those shown in the figure were not representative of the overall results.

The authors also noted that they spliced out lanes from the original image in preparing Fig 2A, and rearranged data in Fig 5A P-AMPKα panel for the purpose of presentation. For both of these panels, the authors provided the original blot data supporting the blots of concern. Individual-level data underlying all the graphs in the article are available.

However, authors have been unable to provide raw unadjusted blots underlying panels of concern in Figs 3A and 6B and are therefore not compliant with our data availability policy.

In light of the image concerns and the unavailability of data underlying the findings, the *PLOS ONE* Editors post this Expression of Concern.
